# Effect of Tongue-Hold Swallow on Pharyngeal Contractile Properties in Healthy Individuals

**DOI:** 10.1007/s00455-020-10217-9

**Published:** 2021-01-01

**Authors:** Yoichiro Aoyagi, Miho Ohashi, Shiori Ando, Yoko Inamoto, Keiko Aihara, Yoko Matsuura, Sayuri Imaeda, Eiichi Saitoh

**Affiliations:** 1 Department of Rehabilitation Medicine, School of Medicine, Fujita Health University, 1-98 Dengakugakubo, Kutsukake, Toyoake, Aichi 470-1192 Japan; 2 Department of Rehabilitation, Fujita Health University Bantane Hospital, Nagoya, Japan; 3 Department of Rehabilitation, Fujita Health University Hospital, Toyoake, Japan; 4 Faculty of Rehabilitation, Fujita Health University, Toyoake, Japan

**Keywords:** Tongue-hold swallow, Superior pharyngeal constrictor, High-resolution manometry, Deglutition, Deglutition disorder

## Abstract

Tongue-hold swallow (THS) is a swallow exercise in which an individual swallows saliva while holding the anterior portion of the tongue between the front teeth. The effect of THS on pharyngeal contractile vigor is still unclear. The purpose of this study was to quantify THS using high-resolution manometry with a contractile integral analysis. Twenty-two healthy participants performed three different saliva swallow tasks: normal swallow, weak THS (in which the tongue was protruded 1 cm outside the upper incisors), and strong THS (in which the tongue was protruded 2 cm outside the upper incisors). The participants repeated each task twice randomly. Pharyngeal and upper esophageal sphincter metrics, including the pharyngeal contractile integral, were analyzed. Both weak and strong THS enhanced the velopharyngeal contractile integral and peak pressure compared with normal swallow (*P* < 0.01). THS also prolonged mesopharyngeal contraction (*P* < 0.01). Holding the tongue anteriorly during swallow requires significant biomechanical changes to pharyngeal contractile properties at the superior and middle pharyngeal constrictor levels; thus, it may serve as a resistance exercise for the muscles that are involved in bolus propulsion.

## Introduction

Tongue-hold swallow (THS), which is also referred to as tongue-hold maneuver/Masako maneuver, is a saliva swallowing exercise that was proposed by Fujiu et al. [1, 2]. In THS, a patient swallows saliva while holding the anterior portion of the tongue between the front teeth. THS focuses specifically on pharyngeal contraction by physiologically increasing the anterior movement of the pharyngeal musculature, thus contributing to improved contact between the tongue base and the posterior pharyngeal wall during the pharyngeal stage of swallowing [1].

Previous studies have addressed the physiological aspects of THS using videofluoroscopy (VF), electromyography, tongue pressure sensors, and manometry. After the early reports by Fujiu et al. using VF, in which anterior bulging of the posterior pharyngeal wall increases by THS [1, 2], few studies have reported the effect of the THS on structural movements during swallowing [3, 4]. A study that used electromyography suggested that the magnitude and duration of submental, genioglossus, and superior pharyngeal constrictor muscle activity increased during THS [5]. Another study also indicated that submental muscle activity increased during THS [6]. Moreover, the duration of tongue pressure generation increased in the posterior circumferential parts of the hard palate during this exercise [6, 7].

Contact between the tongue base and the posterior pharyngeal wall during the pharyngeal stage of swallowing generates swallow-related pressure, which plays an important role in the passage of the bolus through the hypopharynx [8]. Lazarus et al. reported that mesopharyngeal pressure increased during THS in three patients with head and neck cancer [9]. The amplitude and duration of pharyngeal peak pressure was reduced during THS in a study of healthy subjects who used conventional pressure sensors [10]. Umeki et al. used high-resolution manometry (HRM) to identify a lack of differences in the velopharyngeal and mesopharyngeal pressures in the presence or absence of THS. Hammer also reported that the manometric pressure and duration remained unchanged [5]. Thus, although the THS was originally designed to increase posterior pharyngeal wall movement and is widely used in dysphagia rehabilitation, the effect of THS on pharyngeal pressure remains controversial. Therefore, there is a need to understand whether and how swallow-related pressure may change during THS. One possible explanation for this controversy is that the main outcome measures used in previous reports were contractile peak pressures, which have the practical advantage of being easy to determine without the use of highly specialized software. However, peak pressure only indexes one aspect of the phenomenon. In contrast, the contractile integral, which defines pressure over space and time, has gained popularity as a measure of the ‘vigor’ of the pharyngeal swallowing response [11]. Altered pharyngeal contractile integrals associated with aging, different volumes, or swallowing maneuvers have been reported [12–14]. In this study, we quantified the effects of THS on pharyngoesophageal function using HRM and a novel, objective pressure-flow analysis. We hypothesized that the pharyngeal contractile integral measured by the pressure-flow analysis is enhanced with THS.

## Methods

### Participants

Twenty-two healthy participants (15 males and 7 females) participated in the study. Participants were recruited based on written and verbal advertisements. The age range of the patients was 24–56 years, with a mean [± standard deviation (SD)] of 30.3 ± 5.8 years. No participant had a history of pulmonary or neurological disease; structural or speech disorders; or voice, mastication, or swallowing problems. The experiments were approved by the Ethics Committee of Fujita Health University (HM16-215), and written informed consent was obtained from each participant after explanation of the aim and methodology of the study.

### Experimental Setting

All participants were seated comfortably on a chair with their head unsupported. An HRM catheter (PD1236K; Star Medical, Tokyo, Japan) with 36 unidirectional pressure sensors was inserted transnasally through the upper esophagus and into the proximal esophagus. The manometric catheter was lubricated with K-Y™ Jelly (Johnson & Johnson, New Brunswick, NJ, USA) to ease the passage of the catheter through the pharynx. Adjacent sensors were placed 1 cm apart. The HRM manometry catheter had a length of 130 cm and diameter of 4 mm. The sensors are capable of circumferential pressure detection, with an accurate pressure-recording range from − 50 to 300 mm Hg. The pressure data measured by the manometer were amplified and entered into the electrode junction box (GMMS-1000; Star Medical). The data were recorded using a sampling frequency of 40 Hz and uploaded to a Windows-based PC on a real-time basis. A video camera (HD Pro Webcam C920r; Logicool, Tokyo, Japan) was used to monitor the position of the tongue.

### Experimental Protocol

**Fig. 1:**
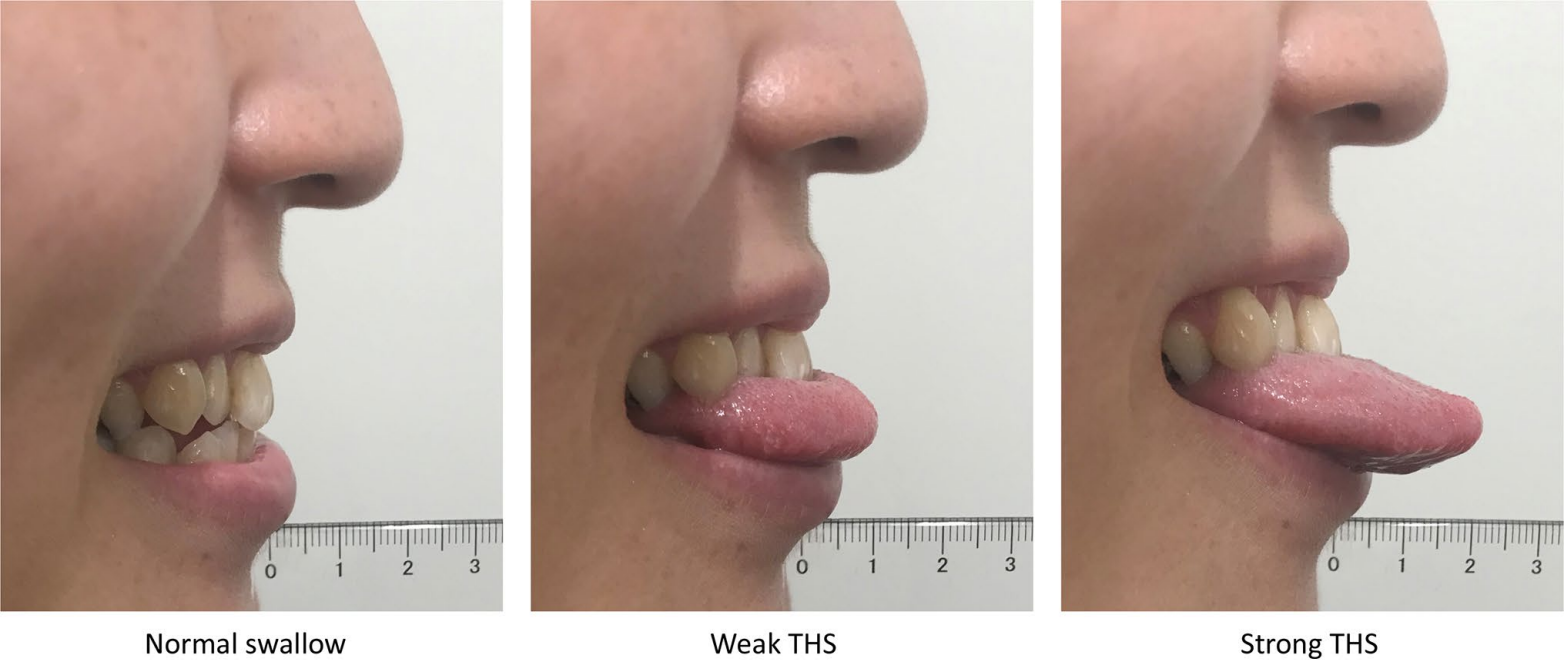
Tongue positions with normal saliva swallow, weak tongue-hold swallow (THS), and strong THS The tongue was protruded 1 and 2 cm outside the upper incisors in weak and strong THS, respectively. The extent of tongue protrusion was adjusted using a transparent scale

Participants performed three types of saliva swallow [7]: (1) normal swallow, in which the tongue was not protruded; (2) weak THS, in which the tongue was protruded 1 cm outside the upper incisors); and (3) strong THS, in which the tongue was protruded 2 cm outside the upper incisors. The extent of tongue protrusion was adjusted using a transparent scale (Fig. 1). Participants were provided enough practice time to familiarize themselves with the THS in advance. For THS, participants were instructed to protrude the tongue from the mouth, hold the anterior portion of the tongue gently between the front teeth or gums, and swallow saliva while keeping the tongue protruded [1, 15]. No specific instructions were given regarding the manner of swallowing (e.g. how to move the tongue or how to push saliva) or to examine the natural changes in swallowing physiology with and without THS. Participants repeated each task twice in a random manner. The randomization was performed by an examiner by drawing out a card from a total of six cards arranged in a random and blinded order. The time interval between the tasks was at least 60 s.

### Data Collection and Analysis

Swallowing events were extracted and analyzed using an adjunctive semiautomated software (Starlet stealth; Star Medical, Tokyo) that located the areas of interest and calculated the requisite metrics of pharyngeal and upper esophageal sphincter (UES) pressure [16]. After the area of a swallowing event was identified upon pressure topography, velopharyngeal, mesopharyngeal, hypopharyngeal, and UES regions were traced manometrically according to McCulloch et al. [17, 18]. The velopharynx includes the two or three most superior sensors with typical bimodal swallowing-related pressure waveforms. The mesopharynx is the area of swallow-related pressure change, with a high-pressure zone located approximately midway between the velopharynx and the UES, with its epicenter at the high-pressure point and extending 2 cm proximal and distal to that point. The hypopharynx is located between the mesopharynx and the UES. Guided by the landmarks of the velopharyngeal, mesopharyngeal, and hypopharyngeal regions, the software then automatically generated the locations and values of peak velopharyngeal, mesopharyngeal, and hypopharyngeal pressures and the onset and offset of the elevated pressures. The pharyngeal contractile duration was defined as the time from the onset to the offset of the elevated pressures.

The UES is the midpoint of stable high pressure located just proximal to the baseline low esophageal pressure zone, extending to a point of low esophageal pressure distally and low baseline pharyngeal pressure proximally. The UES undergoes an elevation of 2 cm or more before complete UES relaxation [19], while the manometry catheter is elevated 1 cm or more during swallowing, asynchronous to UES elevation [19, 20]. Therefore, UES pressure data were analyzed within an area of interest corresponding to the region from the distal margin of the UES high-pressure zone to the estimated apogee position of the UES during the swallow [21]. The maximum axial UES pressure during the swallow was measured within the limits of the UES area of interest over time. The location of the maximum axial pressure was used to track the superior and inferior movement of the UES based on the method of Ghosh et al. [22]. Consecutive pressure values mapped to the corresponding position of the UES over time were used to derive an optimal profile of pressure. UES relaxation was defined as the UES pressure interval that was below 50% of the baseline or 35 mmHg, whichever is lower [23]. The maximum limit of the half-baseline pressure was set at 35 mm Hg if participants had a UES baseline pressure > 70 mm Hg [11, 24]. The maximum UES pressures preceding and succeeding UES relaxation were defined as the maximum pre-opening UES pressure (pre-UES pressure) and the maximum post-closure UES pressure (post-UES pressure). The nadir UES pressure, i.e., the lowest UES pressure during relaxation, was also measured.

Velopharyngeal, mesopharyngeal, hypopharyngeal, and whole pharyngeal contractile integrals and the UES integrated relaxation pressure were analyzed using a semi-automated analysis portal (Swallow Gateway™) [25–27]. These pharyngoesophageal integral metrics are in line with the International High-Resolution Pharyngeal Manometry Working Group recommendations [11]; the metrics used in this study are defined in Table 1. HRM data were uploaded onto Swallow Gateway™. This portal has excellent inter- and intra-rater reliability [28, 29]. Once the data were uploaded onto the web-based application (www.http//swallowgateway.com), labeled swallows were identified and six landmarks (including the position of the velopharynx upper margin, hypopharynx upper margin, UES apogee, and UES distal margin, as well as the onset and offset of UES relaxation) were created, which led to the generation of outcomes [26, 30]. Specific numbers were assigned for all swallowing events; therefore, the researchers who analyzed the data were blinded with regard to the swallow type (normal, weak THS, and strong THS).

**Table 1:** Definitions of pharyngoesophageal integral metrics used in this study

Metric	Unit	Definition
Velopharyngeal contractile Integral	mmHg cm s	Measure of contractile vigor within a space–time box on the pressure topography plot spanning the velopharyngeal region only
Mesopharyngeal contractile integral	mmHg cm s	Measure of contractile vigor within a space–time box on the pressure topography plot spanning the mesopharyngeal region only
Hypopharyngeal contractile integral	mmHg cm s	Measure of contractile vigor within a space–time box on the pressure topography plot spanning the hypopharyngeal region only
Whole pharyngeal contractile integral	mmHg cm s	Global measure of pharyngeal contractile vigor within a space–time box on the pressure topography plot spanning from the velopharynx superiorly to the upper margin of the UES
UES integrated relaxation pressure	mmHg	Measure of the extent of UES relaxation. UES integrated relaxation pressure is the median of the lowest non-consecutive 0.20–0.25 s of e-sleeve pressure

*UES* upper esophageal sphincter

### Statistical Analysis

To evaluate the differences in metrics of pharyngeal and UES pressure and timing between normal swallow, weak THS, and strong THS, we used one-way repeated-measures ANOVA. Greenhouse–Geisser correction was used when the assumption of sphericity was not met. Pairwise comparisons were performed using Bonferroni's multiple comparison test. Statistical analysis was performed using IBM SPSS Statistics (version 26; IBM Japan, Tokyo, Japan). The first and second trials for each task (normal swallow, weak THS, or strong THS) were chosen as a single sample. All values were expressed as means ± SD, and statistical significance was set at *P* < 0.05.

## Results

**Fig. 2:**
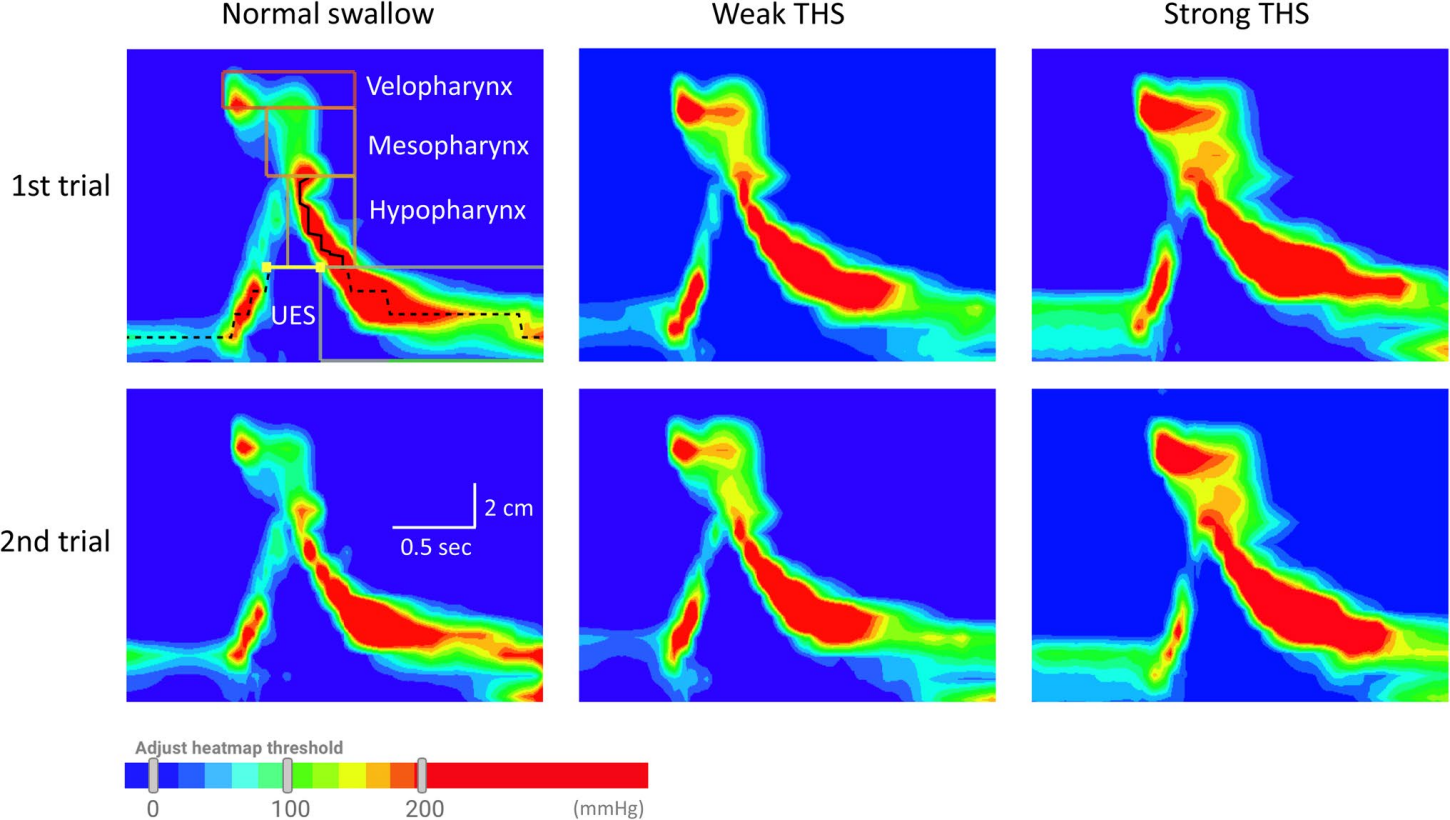
Example of spatiotemporal plots of normal saliva swallow, weak tongue-hold swallow (THS), and strong THS from a single participant, who exhibited differences in velopharyngeal and mesopharyngeal pressure topographies. *UES* upper esophageal sphincter

Examples of three types of saliva swallow with and without THS from a single participant are shown in Fig. 2. The values of pharyngeal and UES metrics with descriptive statistics are shown in Table 2. In the velopharynx, there was a significant main effect of the three types of saliva swallow on peak pressure (F[2, 78] = 18.735, *P* < 0.001) and contractile integral [F(2, 76)  = 11.384, *P* = 0.001]. Significantly higher velopharyngeal peak pressures were generated in weak and strong THS (170 ± 61 and 171 ± 72 mmHg, respectively) compared with normal swallow (140 ± 61 mmHg). Velopharyngeal contractile integrals were significantly increased in weak and strong THS (61 ± 36 and 75 ± 57 mmHg/cm/s) compared with normal swallow (40 ± 32 mmHg/cm/s).

**Table 2:** Summary statistics for pressure metrics recorded during saliva swallowing using normal swallow, weak tongue-hold swallow (THS), and strong THS

Region	Metric	Normal swallow	Weak THS	Strong THS	Bonferroni's multiple comparison test			ANOVA
					Normal swallow vs. Weak THS	Normal swallow vs. Strong THS	Weak THS vs. Strong THS	
Velopharynx	Peak pressure (mmHg)	140 ± 61	170 ± 61	171 ± 72	< 0.001	< 0.001	1.000	< 0.001
	Contractile duration (ms)	597 ± 91	615 ± 92	617 ± 110	0.807	0.656	1.000	0.347
	Contractile integral (mmHg cm s)	40 ± 32	61 ± 36	75 ± 57	< 0.001	0.002	0.221	0.001
Mesopharynx	Peak pressure (mmHg)	208 ± 59	231 ± 57	227 ± 68	0.134	0.325	1.000	0.075
	Contractile duration (ms)	388 ± 89	435 ± 106	430 ± 127	0.002	0.046	1.000	0.004
	Contractile integral (mmHg cm s)	106 ± 47	127 ± 67	128 ± 85	0.097	0.251	1.000	0.073
Hypopharynx	Peak pressure (mmHg)	205 ± 80	224 ± 80	232 ± 91	0.106	0.061	0.557	0.020
	Contractile duration (ms)	340 ± 101	363 ± 109	336 ± 96	0.790	1.000	0.056	0.244
	Contractile integral (mmHg cm s)	138 ± 60	162 ± 99	148 ± 90	0.166	0.789	0.557	0.081
Whole pharynx	Contractile integral (mmHg cm s)	331 ± 125	390 ± 186	389 ± 193	0.028	0.031	1.000	0.008
UES	Nadir pressure (mmHg)	− 4 ± 10	− 5 ± 11	− 6 ± 11	0.943	0.711	1.000	0.132
	Maximum pre-UES pressure (mmHg)	164 ± 70	86 ± 32	92 ± 44	1.000	1.000	1.000	0.395
	Maximum post-UES pressure (mmHg)	289 ± 100	288 ± 83	294 ± 95	1.000	1.000	1.000	0.878
	Integrated relaxation pressure (mmHg)	4 ± 14	7 ± 21	5 ± 17	0.896	1.000	1.000	0.466
	Relaxation duration (ms)	396 ± 86	432 ± 103	416 ± 109	0.198	0.711	1.000	0.132

Values are presented as the average ± standard deviation

*UES* upper esophageal sphincter, *pre-UES* pre-opening UES, *pre-UES* pre-opening UES, *versus* vs

In the mesopharynx, there was a significant main effect on contraction duration [F(2, 78) = 6.014, *P* = 0.004]. A significantly longer mesopharyngeal contraction pressure was generated during weak and strong THS (435 ± 106 and 430 ± 127 mmHg) compared with normal swallow (388 ± 89 mmHg). Regarding the whole pharyngeal contractile integral, there was a significant main effect [F(2, 76) = 5.161, *P* = 0.008]. Significantly longer mesopharyngeal contraction durations were generated in weak and strong THS (435 ± 106 and 430 ± 127 ms) compared with normal swallow (388 ± 89 ms). Hypopharyngeal and UES metrics were not affected by the three types of saliva swallow.

## Discussion

This study was the first to evaluate THS using HRM with a novel, objective pressure-flow analysis. Some important findings were obtained. First, THS enhanced the velopharyngeal contractile vigor, which was in accordance with our hypothesis. Second, THS prolonged the mesopharyngeal contraction duration. Third, weak and strong THS contributed equally to the biomechanical changes in velopharyngeal contractile vigor and mesopharyngeal contraction duration.

Anatomically, soft palate elevation and superior pharyngeal constriction generate velopharyngeal pressure [31]. Fibers of the superior pharyngeal constrictor muscle connect with fibers of the posterior portion of the transverse lingual muscle and form a ring of muscle [32]. The genioglossus muscle is active during protrusive tongue movement, while transverse lingual muscle is active during retrusive tongue movement, as an antagonist of the genioglossus muscle [33]. In THS, the activity of genioglossus and superior pharyngeal constrictor muscles is increased significantly [5]. Therefore, it seems reasonable that the ring of muscle formed by the transverse lingual muscle and the superior pharyngeal constrictor is highly responsible for the increment of the posterosuperior movement of the tongue and the constrictive movement of the pharyngeal cavity during THS, thus contributing to the enhancement of the velopharyngeal contractile activity. In contrast with our findings, Hammer et al. did not find any difference in velopharyngeal pressures between normal swallow and THS [5]. There are two possible explanations for these observations. One is the method used for pressure analysis; this study investigated not only peak pressure or pressure duration but also contractile integral metrics, which display more multimodal and/or sustained features. The velopharynx typically includes bimodal swallowing-related pressure waveforms. Although an added value of the pharyngeal contractile integral as compared with peak pressure was not proven, the contractile integral may have greater value for recording pressure within the velopharyngeal region in particular [11]. Another reason is the difference in the size of the samples used; Hammer et al. targeted eight subjects, whereas this study targeted 22 subjects.

In this study, the duration of mesopharyngeal contraction was prolonged during THS without an increment in pressure. Fujiu and Logemann observed the anterior bulging of the posterior pharyngeal wall during THS at the mid and inferior C2 level, which corresponds to the middle pharyngeal constrictor [1]. Submental, genioglossus, and superior pharyngeal constrictor muscle activity increases significantly in THS [5][5]. Thus, in the presence of a restricted tongue retrusive movement while the tongue is held anteriorly, THS may require stronger and longer pharyngeal swallowing without a pressure increment at the mesopharynx using the middle pharyngeal constrictor. Thus, THS is likely to serve as a resistance exercise for the muscles that are involved in bolus propulsion. A larger protrusion of the tongue during THS will render the initiation of swallowing more difficult. Nevertheless, we did not find any difference in pharyngeal or UES pressure metrics according to the extent of tongue protrusion. The reason for this remains unknown. As the tongue base retraction is reduced, the bulging of the pharyngeal wall increases. A possible explanation for this is that these phenomena might occur in approximately equal proportions as the tongue is protruded > 1 cm outside the upper incisors. In other words, a ceiling effect might be observed for protrusions > 1 cm.

A limitation of this study may be that we did not consider the individual differences in tongue flexibility (i.e. maximum tongue protrusion length) [6, 7]. The individual tongue flexibility might specify the amount of load placed on the tongue and reveal physiological phenomena in greater detail. Finally, we acknowledge that, at present, we are unable to determine whether the THS would serve well as a resistance exercise for tongue or pharyngeal muscles, as we simply examined each subject during a single test session. Our findings suggest that there is a reasonable physiological rationale on which to base future clinical studies. The next steps of this investigation will be to examine the THS in specific patients with clinical dysphagia and to determine the exercise strengths and schedules that may be of clinical benefit. A study of THS including manometric, electromyographic, and imaging modalities is desirable in clinical populations.
